# Phylogenetic indices and temporal and spatial scales shape the neighborhood effect on seedling survival in a mid‐mountain moist evergreen broad‐leaved forest, Gaoligong Mountains, Southwestern China

**DOI:** 10.1002/ece3.11675

**Published:** 2024-07-07

**Authors:** Liping Wang, Junjie Wu, Yong Chai, Jiwen Sun, Xiaoli Yu, Zhe Feng, Fengxian Chen

**Affiliations:** ^1^ College of Agriculture and Biological Science Dali University Dali Yunnan China; ^2^ Yunnan Academy of Forestry and Grassland Kunming China; ^3^ Yunnan Key Laboratory of Biodiversity of Gaoligong Mountain Kunming China; ^4^ Gaoligong Mountain Forest Ecosystem Observation and Research Station of Yunnan Province Kunming China

**Keywords:** Gaoligong Mountains, habitat filtering, neighborhood effects, phylogenetic relatedness, seedling survival

## Abstract

Density dependence and habitat filtering have been proposed to aid in understanding community assembly and species coexistence. Phylogenetic relatedness between neighbors was used as a proxy for assessing the degree of ecological similarity among species. There are different conclusions regarding the neighborhood effect in previous studies with different phylogenetic indices or at different spatiotemporal scales. However, the effects of density dependence, neighbor phylogenetic relatedness, and habitat filtering on seedling survival with different phylogenetic indices or at different temporal and spatial scales are poorly understood. We monitored 916 seedlings representing 56 woody plant species within a 4‐ha forest dynamics plot for 4 years (from 2020 to 2023) in a subtropical mid‐mountain moist evergreen broad‐leaved forest in the Gaoligong Mountains, Southwestern China. Using generalized linear mixed models, we tested whether and how four phylogenetic indices: total phylogenetic distance (TOTPd), average phylogenetic distance (AVEPd), relative average phylogenetic distance (APd′), and relative nearest taxon phylogenetic distance (NTPd′), three temporals (1, 2, and 3 years), and spatial scales (1, 2, and 4 ha) affect the effect of density dependence, phylogenetic density dependence, and habitat filtering on seedling survival. We found evidence of the effect of phylogenetic density dependence in the 4‐ha forest dynamics plot. The effects of density dependence, phylogenetic density dependence, and habitat filtering on seedling survival were influenced by phylogenetic indices and temporal and spatial scales. The effects of phylogenetic density dependence and habitat filtering on seedling survival were more conspicuous only at 1‐year intervals, compared with those at 2‐ and 3‐year intervals. We did not detect any effects of neighborhood or habitat factors on seedling survival at small scales (1 and 2 ha), although these effects were more evident at the largest spatial scale (4 ha). These findings highlight that the effects of local neighborhoods and habitats on seedling survival are affected by phylogenetic indices as well as temporal and spatial scales. Our study suggested that phylogenetic index APd′, shortest time scale (1 year), and largest spatial scales (4 ha) were suitable for neighborhood studies in a mid‐mountain moist evergreen broad‐leaved forest in Gaoligong Mountains. Phylogenetic indices and spatiotemporal scales have important impacts on the results of the neighborhood studies.

## Graphical abstract

We tested whether and how four phylogenetic indices (TOTPd, AVEPd, APd′, and NTPd′) and three temporals (1, 2, and 3 years) and spatial scales (1, 2, and 4 ha) affect the effect of DD, phylogenetic density dependence (PDD), and habitat filtering on seedling survival by using generalized linear mixed models. We found that the phylogenetic indices did affect the effect of PDD and habitat filtering on seedling survival. The fitting results of models on seedling survival were inconsistent at different spatiotemporal scales.

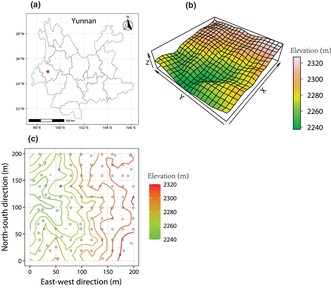

## 1 INTRODUCTION

The neighborhood effect is considered an important factor in illustrating the patterns of tree survival and growth (Comita et al., 2009).

Density dependence is defined as individuals having low survival, growth, and recruitment, and high mortality when surrounded by a high density of neighbors. Conspecific negative density dependence indicates that seedling survival can decrease near conspecific individuals (Connell, 1971; Janzen, 1970).

Recently, the majority of studies have shown that the phylogenetic relatedness of neighbors is a vital predictor of density dependence (Metz et al., 2010; Webb et al., 2002, 2006; Wu et al., 2016; Zhu et al., 2015). Accordingly, phylogenetic density dependence is an extension of density dependence. Webb et al. (2002) proposed the concept of phylogenetic dependence for the first time based on the accumulation of long‐term research in ecology combined with evolutionary biology, and Webb et al. (2006) confirmed that seedling survival was enhanced by a neighborhood where in heterospecifics are not closely related. Thus, it is reasonable to consider the phylogenetic relatedness of heterospecific neighbors when assessing neighborhood effects (Comita et al., 2018; Webb et al., 2006; Zhu et al., 2015).

Closely related species may exert a negative density dependence owing to niche overlap, which increases resource competition between seedlings and surrounding species (Burns & Strauss, 2011; Chen et al., 2016; Du et al., 2017; Metz et al., 2010; Zhu et al., 2015), and increases the possibility of being attacked by natural enemies that are attracted by closely related surrounding species (Chen et al., 2018; Gilbert & Webb, 2007; Liu et al., 2012; Paine et al., 2012; Queenborough et al., 2009; Shuai et al., 2014). Thus, the effect is referred to as phylogenetic negative density dependence. In contrast, neighbors with closer phylogenetic distance have similar environmental preferences, and they will aggregate and grow in similar habitats, which is called phylogenetic positive density dependence. Phylogenetic positive density dependence is associated with habitat heterogeneity (Tito de Morais et al., 2020), habitat filtering (Cao et al., 2018; Huang et al., 2022; Lebrija‐Trejos et al., 2014; Wu et al., 2016; Zhu et al., 2015), and symbiosis with mycorrhizal fungi (Jiang et al., 2022). However, contrary to the effects of phylogenetic negative density dependence and phylogenetic positive density dependence, there is no effect of phylogenetic density dependence on seedling survival, which is mainly due to the absence of a phylogenetic signal for functional traits (Kunstler et al., 2012; Lyu et al., 2017; Uriarte et al., 2010) and no direct interaction between focal seeds or seedlings and their neighbors (Williams et al., 2021).

A growing number of phylogenetic indices have been calculated as the phylogenetic distance between neighbors, and have been used in various studies. Gonzalez et al. (2010) found that Faith's phylogenetic diversity (phylogenetic richness), which represents the sum of the branch lengths of the species present in a community (Faith, 1992), drove plant recruitment. In recent years, the net relatedness index (NRI) and nearest taxon index (NTI) (Webb, 2000; Webb et al., 2002), as an indicator of phylogenetic density dependence in species coexistence, have been widely used to study the phylogenetic structure of forest community (Liu et al., 2012; Shuai et al., 2014; Uriarte et al., 2010). Levin et al. (2020) calculated phylogenetic distinctiveness using two metrics (the mean pairwise distance (MPD) and nearest neighbor distance (NND)) to explain alien plant population responses to competition. Lebrija‐Trejos et al. (2014) estimated the phylogenetic distance (Pd) on seedling survival; it is crucial to note that Pd is the mean distance of all neighbors to the focal seedling (Webb et al., 2006), and is not MPD, as used to calculate NRI (Webb et al., 2002). Inconsistent results of phylogenetic density dependence have been obtained for different indices such as NND (Levin et al., 2020), MPD (Levin et al., 2020), NRI, NTI (Liu et al., 2012; Shuai et al., 2014; Zhu et al., 2015), and Pd (Webb et al., 2006) obtained phylogenetic negative density dependence. It is still unclear whether these differences are different results due to different phylogenetic indices.

The seedling stage is the most vulnerable stage in the life history of plants, and seedlings are also the most sensitive to varied environmental factors (Wright, 2002; Wright et al., 2005). Compared to adult trees, seedling survival is strongly influenced by biotic factors (e.g., seedling size and the interactions of conspecific and heterospecific neighborhoods) (Uriarte et al., 2004; Wang et al., 2020), habitat conditions (topography, soil properties, and light), and interannual climate variability (e.g., annual temperature and rainfall) (Altizer et al., 2013; Bachelot et al., 2015; Xu et al., 2022). Consequently, considerable attention has been focused on the seedling stage.

The strengths of density dependence, phylogenetic density dependence, and habitat filtering have been shown to vary spatiotemporally because of spatiotemporal variations in resource availability and recognizable patterns of seedling dynamics (Comita et al., 2009; Lin et al., 2012). The drivers of density dependence, phylogenetic density dependence, and habitat filtering may change over time. Soil organisms (Bardgett et al., 2008), insects (Chaves et al., 2012), and pathogens (Altizer et al., 2013) change over time, which in turn might lead to variations in the effects of neighbors and habitats on seedlings (Bachelot et al., 2015). However, few studies have investigated whether and how the effects of density dependence, phylogenetic density dependence, and habitat factors on seedling survival vary at different temporal and spatial scales. Previous studies found that neighborhood effect and habitat filtering on tree growth exhibited spatial–temporal dependence (Weng et al., 2022), which tended to be more varied with time (Bai et al., 2012). What is more, there has also been demonstrated variance over time in the phylogenetic distance of seedlings at a site (Webb et al., 2006), and the phylogenetic clustering of neighbors varies with spatial variation. As such, it is important to study the effects of density dependence, phylogenetic density dependence, and habitat factors on seedling survival at different temporal and spatial scales.

In this study, we used seedling dynamic data from a mid‐mountain moist evergreen broad‐leaved forest to explore the effects of density dependence, phylogenetic density dependence, and habitat filtering on seedling survival. Seedling data of 56 woody species were collected over 4 years to answer the following questions: (1) Is the strength of the effect of density dependence, phylogenetic density dependence, and habitat filtering in a mid‐mountain moist evergreen broad‐leaved forest in the Gaoligong Mountains different in models with different phylogenetic indices? (2) Does the strength of the effects of density dependence, phylogenetic density dependence, and habitat filtering in a mid‐mountain moist evergreen broad‐leaved forest in the Gaoligong Mountains differ between models at different temporal and spatial scales?

## 2 MATERIALS AND METHODS

### 2.1 Study site and seedling census

**FIGURE 1:**
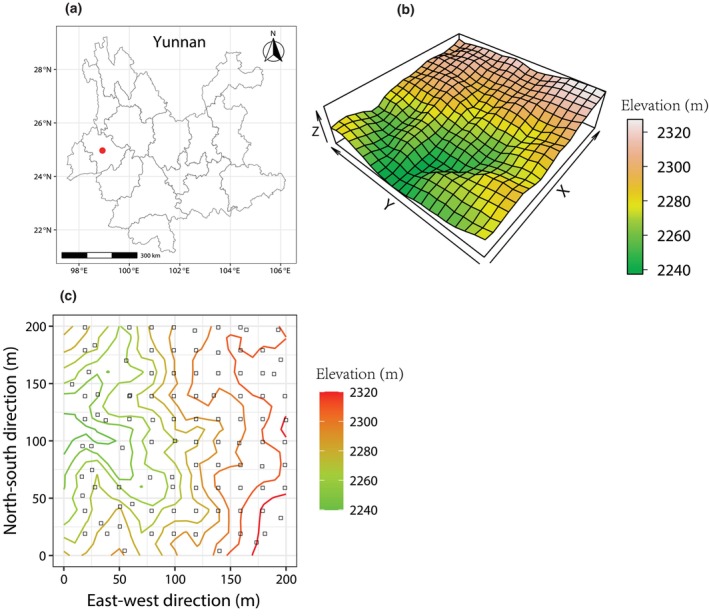
The location and map of the 4‐ha dynamics plot. (a) The location of the 4‐ha dynamics plot in Gaoligong Mountains is indicated with a red dot. (b)The 3D perspective map of the 4‐ha dynamics plot, and (c) the distribution of 100 seedling quadrats (black hollow squares) within the 4‐ha dynamics plot.

The study was conducted in Gaoligong Mountains (24°56′–28°22′ N, 98°08′–98°50′ E), which is located in Yunnan Province, Southwestern China. A 4‐ha (200 m × 200 m) dynamics plot (24°50′9.8″–24°50′17.3″ N, 98°45′53.1″–98°46′1.3″ E) in the southern section of Gaoligong Mountains was established following the construction standard of CForBio in 2009–2010 (Figure 1a). The plot is a subtropical mid‐mountain moist evergreen broad‐leaved forest. It is characterized by a subtropical plateau monsoon with a mean annual temperature of 17°C and a mean annual precipitation of 1200 mm. The rainy season is from May to October and the dry season is from November to April of the following year (Meng et al., 2013). The elevation is between 2135 and 2329.65 m. This plot runs in a north–south direction and is high in the east and low in the west (Figure 1b). Meanwhile, all woody plants with a diameter at breast height (DBH) ≥1 cm were tagged, mapped, measured, and identified to the species level. There were 10,546 woody plants belonging to 95 species, 64 genera, and 35 families. The four dominant species were *Symplocos ramosissima*, *Eurya pseudocerasifera*, *Polyspora longicarpa*, and *Neolitsea lunglingensis*.

A total of 100 seedling quadrats (2 × 2 m) were established in November 2020 and located at the top right of each subplot (20 × 20 m) in the 4‐ha dynamics plot. If these locations of the seedling quadrat were prevented by obstacles such as streams, large trees, rocks, or fallen wood, they were placed in nearby 5 × 5 m subplots (Figure 1c). In each of these seedling quadrats, all woody seedlings with DBH <1 cm and height ≥ 20 cm were tagged, measured, and identified to species. Seedling quadrats were subsequently censused annually from 2021 to 2023. In addition, all new recruits to the 20‐cm‐height threshold were identified and tagged in each census. We monitored 916 seedlings of 56 species.

### 2.2 Neighborhood variables

The adult and seedling neighbors included trees, shrubs, and lianas, but focal seedlings did not include liana species. Conspecific seedling neighbors (S_con) were defined as the number of conspecific seedling neighbors within the same seedling quadrat as the focal seedling. The densities of conspecific adult trees (A_con) within a 20 m radius were calculated as follows:

(1) $$A\_\mathrm{con}=\sum_{i}^{N}\frac{{\mathrm{BA}}_{i}}{{\text{Distance}}_{i}}$$

where *N* is the number of adult neighbors. BA_*i*_ is the basal area of the neighboring adult *i*. Distance_*i*_ is the distance between the center of the focal seedling quadrat and adult neighbor *i*.

### 2.3 Construction of phylogenetic trees and phylogenetic indices

To estimate phylogenetic distances between focal seedlings and neighbors, we created a phylogenetic tree for species of this plot using the package “V.PhyloMaker 2” (Jin & Qian, 2022), which was based on the LCVP nomenclature standardization system and an updated and expanded version of the dated megaphylogeny GBOTB (Smith & Brown, 2018).

We used four phylogenetic indices to quantify the phylogenetic distance between focal seedlings and their heterospecific neighbors in our study. The total phylogenetic distance (TOTPd), average phylogenetic distance (AVEPd), relative average phylogenetic distance (APd′), and relative nearest taxon phylogenetic distance (NTPd′) were calculated for both seedlings and adult tree neighbors (Webb et al., 2006; Wu et al., 2016). Four phylogenetic indices were calculated as follows:

(2) $$\text{TOTPd}=\sum_{1}^{i}X_{i}\times {\text{abundance}}_{i}$$

(3) $$\text{AVEPd}=\frac{\sum_{1}^{i}X_{i}\times {\text{abundance}}_{i}}{\sum_{1}^{i}{\text{abundance}}_{i}}$$

(4) $$\mathrm{AP}d^{\prime }=\frac{\text{AVEPd}-\text{mean}\left({\text{AVEPd}}_{\text{null}}\right)}{\mathrm{SD}\left({\text{AVEPd}}_{\text{null}}\right)}$$

(5) $${\text{NTPd}}^{\prime }=\frac{\text{MINPd}-\text{mean}\left({\text{MINPd}}_{\text{null}}\right)}{\mathrm{SD}\left({\text{MINPd}}_{\text{null}}\right)}$$

where *X*_*i*_ is the phylogenetic distance between species *i* and the focal seedling species; abundance_*i*_ is the number of neighbors (adults or seedlings) of species *i*. AVEPd_null_ and MINPd_null_, respectively, are the observed AVEPd and MINPd (the minimum of the phylogenetic distance between the focal individual and the heterospecific neighbor) in the null model that shuffled species on phylogeny 999 randomly to generate a null distribution of phylogenetic diversities.

Values of APd′ and NTPd′ > 0 indicate that neighbors are less related to focal seedlings than expected under the null model (higher phylogenetic diversity), and values <0 indicate that neighbors are more closely related than expected via the null model (lower phylogenetic diversity). Larger values of TOTPd, AVEPd, APd′, and NTPd′ indicate the less close phylogenetic distance between the focal seedling and neighbors.

### 2.4 Habitat variables

To account for the effects of environmental variables on seedling survival, we measured habitat variables, including topography, soil properties, and canopy openness.

#### 2.4.1 Topography

The topographic variables in this study included elevation, convexity, and slope following Harms et al. (2001). Elevation was obtained as the mean elevation at the four corners of the seedling quadrat. Convexity was defined as the elevation difference between the focal quadrat and the mean elevation of the eight neighboring quadrats, whereas the convexity of quadrats on the edges of the plot was calculated by subtracting the mean of the four corners and elevations of the surrounding subplots from the elevation at its center. The slope was measured as the mean angular deviation from the horizontal plane of each of the four triangular planes by connecting the three corners separately.

#### 2.4.2 Soil properties

Ten soil variables were measured during the summer of 2022: soil pH, electrical conductivity (EC), organic matter content (C), available phosphorus (AP), available potassium (AK), total nitrogen (TN), total phosphorus (TP), total potassium (TK), soil temperature, and moisture, following the protocols of John et al. (2007).

First, three sampling points were randomly selected from each seedling quadrat, and dead branches or leaves on the surface layer of the ground were removed. The soil temperature and moisture content were measured using a soil hygrothermograph (YDSC A01) at a depth of 5 cm. The mean soil temperature and moisture in each seedling quadrat were used. A quick nitrogen, phosphorus, and potassium meter (TRREC N01, Shandong Sean) was inserted into the soil surface at a depth of 0–5 cm to measure the soil pH, EC, AP, and AK.

In addition, we collected approximately 500 g of 0–5 cm surface soil with a ring knife (5 cm long) at three sampling points for each seedling quadrat. A total of 300 fresh soil samples were collected, packed into bags, and taken to the laboratory for air drying. Subsequently, we determined the organic carbon content (the volumetric of sulfuric acid–potassium dichromate oxidation method, NY/T 1121.6‐2006), total nitrogen (automatic nitrogen determination method, NY/T 1121.24‐2012), total phosphorus (molybdenum antimony anti‐colorimetric method, LY/T 1232‐2015), and total potassium content (hydrofluoric acid and perchloric acid cooking flame photometer method, LY/T 1234‐2015) in these soil samples.

#### 2.4.3 Canopy openness

To assess the light conditions in each seedling quadrat, the canopy openness was quantified using the gap light index (GLI). Hemispherical photographs were taken using a round fisheye lens (12 mm F2.8, Senyo, South Korea) mounted on a Nikon camera (Z5N1933, Nikon, Japan) placed 1.3 m aboveground at the center of each quadrat in December 2021. The photographs were analyzed using Gap Light Analyzer software following Ridler and Calvard (1978) to calculate the canopy openness for each seedling quadrat.

To reduce the number of parameters in the model, principal component analysis (PCA) was performed to assuage the collinearity of the above‐tree topographic variables and the soil variables using the prcomp function in the “stats” package. The light environment data were not included in PCA and were directly incorporated into the models (Comita et al., 2009; Queenborough et al., 2009; Wu et al., 2016). The first three principal components accounted for 66.15% of the variation in the 13 habitat variables. The first principal component (PCA1) was associated with high elevation, EC, C, AP, AK, TN, TP, soil moisture, and low TK. The second principal component (PCA2) was associated with high C and TN and low EC, AP, AK, TK, and soil moisture. The third component (PCA3) was associated with low elevation, convexity, slope, and soil pH (Table S1).

### 2.5 Statistical analysis

We conducted separate analyses for all living seedlings at four 1‐year intervals (2020–2023). Generalized linear mixed models with binomial error distributions were constructed and used the R package lme4 (Bates et al., 2015) to analyze the effects of neighbor densities, phylogenetic relatedness, and habitat factors on seedling survival. The survival of seedlings per year was regarded as the response variable and expressed as 1 (surviving) or 0 (dead). Seedling height was log‐transformed, and then all continuous variables were standardized using the mean and standard deviation of this variable for normalization and divided by 1 SD before analysis, which allowed us to directly compare the relative importance of these explanatory variables (Gelman & Hill, 2006). The means and ranges of all continuous explanatory variables used in the analysis are listed in Table S2.

We constructed phylogenetic + habitat models (see Appendix S1 for a detailed description), including the height of the focal seedlings, the density of conspecific seedlings and adult neighbors, phylogenetic relatedness of the focal seedling and heterospecific seedlings and adult neighbors, and habitat variables as fixed effects at 1‐year intervals. Seedling quadrats were regarded as having random effects in the models to exclude spatial autocorrelation. Furthermore, we included species identities as a random effect because seedlings of different species were expected to respond differently to local neighborhood variables (Lin et al., 2012).

The Akaike's information criterion (AIC) was used to compare candidate models, of which the minimum ΔAIC (the maximum AIC − minimum AIC of models) value was the best model, and the models with a ΔAIC ≤2 were equally judged valid.

To estimate whether there were differences in the effects of different phylogenetic indices on seedling survival, we used S_con and A_con as indicators of conspecific density dependence, used TOTPd, AVEPd, APd′, and NTPd′ of heterospecific neighbor seedlings and adults as indicators of phylogenetic density dependence, respectively, and used light, topography, and soil properties as indicators of habitat conditions in phylogenetic + habitat models. Sequentially, we established phylogenetic + habitat models with the best phylogenetic index at three temporal scales: 1‐year interval (2020–2021, 2021–2022, and 2022–2023), 2‐year interval (2020–2022 and 2021–2023), and 3‐year interval (2020–2023); and at three spatial scales: 1 ha (100 × 100 m), 2 ha (200 × 100 m), and 4 ha (200 × 200 m). Phylogenetic + habitat model was selected because it strongly reflects the influence of habitat filtering on the detection of phylogenetic density dependence on seedling survival in forest communities (Cao et al., 2018; Du et al., 2017). The AIC and conditional *R* ^2^ values, that is, variance explained by both fixed and random effects (Nakagawa & Schielzeth, 2013), were used to compare the models using the MuMIn package (Bartoń, 2023).

The estimated coefficients represent the relative strength of the variable effects; coefficients >0 indicate positive effects on seedling survival, whereas coefficients <0 indicate negative effects. Specifically, the estimated coefficient of the phylogenetic distance indices was positive, indicating that the phylogenetic similarity of the heterospecific neighbors was negatively correlated with seedling survival (phylogenetic negative density dependence), and vice versa.

All analyses were performed using R 4.3.2 (R Core Team, 2023).

## 3 RESULTS

### 3.1 The variance of the effects of four phylogenetic indices in the phylogenetic + habitat models

In the 100 focal seedling quadrats, there were 914, 916, 834, and 854 living seedlings of woody plant species in the 2020, 2021, 2022, and 2023 census periods, respectively.

To compare the effects of different phylogenetic indices on seedling survival, we constructed phylogenetic + habitat models with four phylogenetic indices at 1‐year intervals (Table 1) and 2‐year and 3‐year intervals (Table S3). We found differences in the results of the four indices. The model with TOTPd and the minimum AIC was the best fit for the 2020–2021 interval. The ΔAIC values of all four phylogenetic indices were less than 2 in the 2021–2022 interval, and the AIC value of the model with the APd′ indices was minimum. However, the models with APd′ and NTPd′ were equivalent and best in 2022–2023 interval.

**TABLE 1:** Akaike's information criterion (AIC) and corresponding *R* ^2^ for fixed and random effects (*R* ^2^) values for phylogenetic + habitat models for each of three 1‐year intervals.

Models	2020–2021		2021–2022		2022–2023	
	AIC	*R* ^2^	AIC	*R* ^2^	AIC	*R* ^2^
Height + S_con + A_con + S_TOTPd + A_TOTPd + light + PCA1 + PCA2 + PCA3	**229.13**	.049	**336.77**	.061	271.02	.062
Height + S_con + A_con + S_AVEPd + A_AVEPd + light + PCA1 + PCA2 + PCA3	236.27	.068	**336.49**	.070	271.24	.057
Height + S_con + A_con + S_APd′ + A_APd′ + light + PCA1 + PCA2 + PCA3	234.80	.017	**335.93**	.062	**267.00**	.057
Height + S_con + A_con + S_NTPd′ + A_NTPd′ + light + PCA1 + PCA2 + PCA3	237.71	.113	**337.32**	.065	**268.86**	.059

*Note*: A bold font indicates that the ΔAIC ≤ 2. Height of focal seedlings (Height), density of conspecific seedling neighbors (S_con), and density of conspecific adult neighbors (A_con). Environmental variables included canopy openness (light) and the first three principal components (PCA1, PCA2, and PCA3) of the topographic variables and soil properties. Four phylogenetic distance indices: total phylogenetic distance (TOTPd), average phylogenetic distance (AVEPd), relative average phylogenetic distance (APd′), and relative nearest phylogenetic distance (NTPd′), respectively, for seedling neighbors (S_TOTPd, S_AVEPd, S_APd′, and S_NTPd′) and adult neighbors (A_TOTPd, A_AVEPd, A_APd′, and A_NTPd′).

**FIGURE 2:**
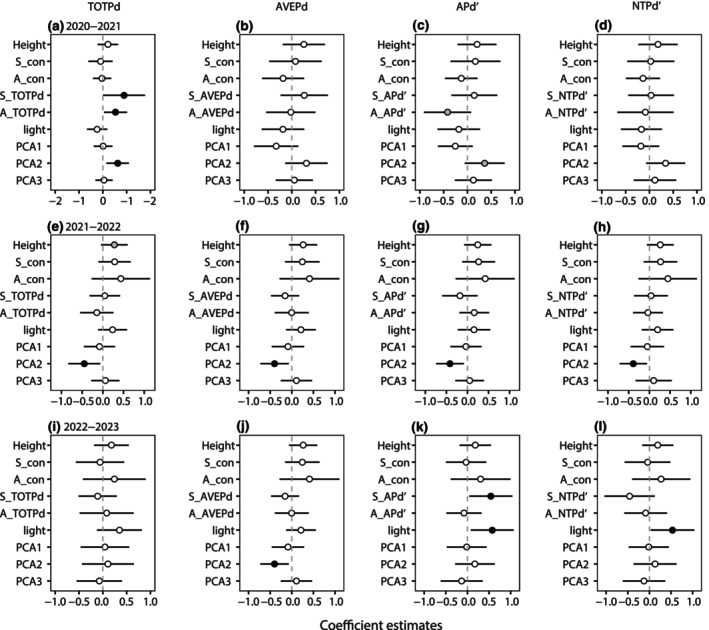
Estimated coefficients (mean ± SE) of neighbor densities and habitat variables on seedling survival using four phylogenetic indices of phylogenetic + habitat models. 95% Confidence intervals of the variables (error bars represent 95% confidence intervals). The black circles indicate significant effects (*p* < .05), gray circles signify marginally significant effects (.05 < *p* < .1), and white circles mean no significance (*p* ≥ .1). See Table 1 for variable abbreviations.

The variance of significances of four phylogenetic indices quantified the effects of phylogenetic relatedness of heterospecific neighbors on seedling survival. We found that seedling and adult TOTPd phylogenetic indices had a positive effect on seedling survival during the 2020–2021 interval (Figure 2a, Table S4). The effect of adult APd′ on seedling survival was marginally negatively significant (Figure 2c) in the 2020–2021 interval, and seedling APd′ strongly positively affected seedling survival in the 2022–2023 interval (Figure 2k). AVEPd and NTPd did not differ significantly at any interval.

There was no significant effect of seedling height or neighbor density (conspecific seedlings and adults) on seedling survival at almost all intervals and phylogenetic indices (Figure 2, Figure S1, Table S4). Apart from height, TOTPd had a marginally positive effect on seedling survival during 2021–2022 (Figure 2e). Seedling survival in the habitats varied significantly among the four indices. The second principal component of topographic variables and soil properties (PCA2), respectively, was significant and marginally significantly positive on seedling survival in the models with TOTPd and APd′ for 2020–2021 interval (Figure 2a,c). However, there was a significant negative effect of PCA2 on seedling survival in all phylogenetic indices for the 2021–2022 interval, and this effect was observed only in the AVEPd models for the 2022–2023 interval (Figure 2g). The first (PCA1) and third (PCA3) principal components of topographic variables and soil properties were not significant for any phylogenetic indices. In addition, we found evidence that light had significant positive effects on seedling survival in models with APd′ and NTPd′ for 2022–2023 interval (Figure 2k,l). Therefore, the APd′ index was selected for subsequent comparisons.

### 3.2 The variance of the effects of three temporal scales in the phylogenetic + habitat models

**FIGURE 3:**
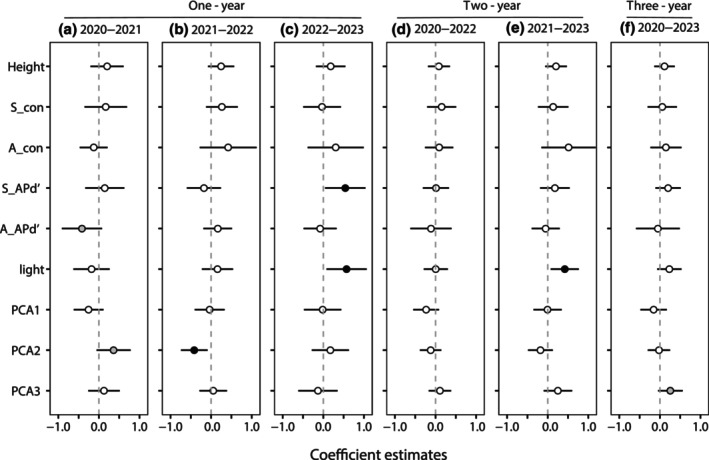
Estimated coefficients (mean ± SE) of neighbor densities and habitat variables on seedling survival of phylogenetic + habitat models at three spatiotemporal scales. 95% Confidence intervals of the variables. The black circles indicate significant effects (*p* < .05), gray circles signify marginally significant effects (.05 < *p* < .1), and white circles mean no significance (*p* ≥ .1). See Table 1 for variable abbreviations.

The effects of phylogenetic density on seedling survival varied between the intervals. In the 1‐year intervals, adult APd′ was marginally significant in the 2020–2021 interval (Figure 3a, Table S4), and seedling APd′ was significant for seedling survival in 2022–2023 (Figure 3c). Conversely, there were no significant effects of phylogenetic density on seedling survival in the 2‐year intervals and the 3‐year interval (Figure 3d–f). Compared to longer intervals, the effect of phylogenetic density on seedling survival was more obvious at short intervals. Height, density of conspecific seedling and adult neighbors, and PCA1 did not significantly affect seedling survival at any of the time points. Light was beneficial for seedling survival during the 2022–2023 and 2021–2023 intervals (Figure 3c,e). PCA2 was significant only in the 2021–2022 interval. Notably, PCA3 had a marginally significant positive effect on seedling survival at the 3‐year interval (Figure 3f).

### 3.3 The variance of the effects of three spatial scales in the phylogenetic + habitat models

**FIGURE 4:**
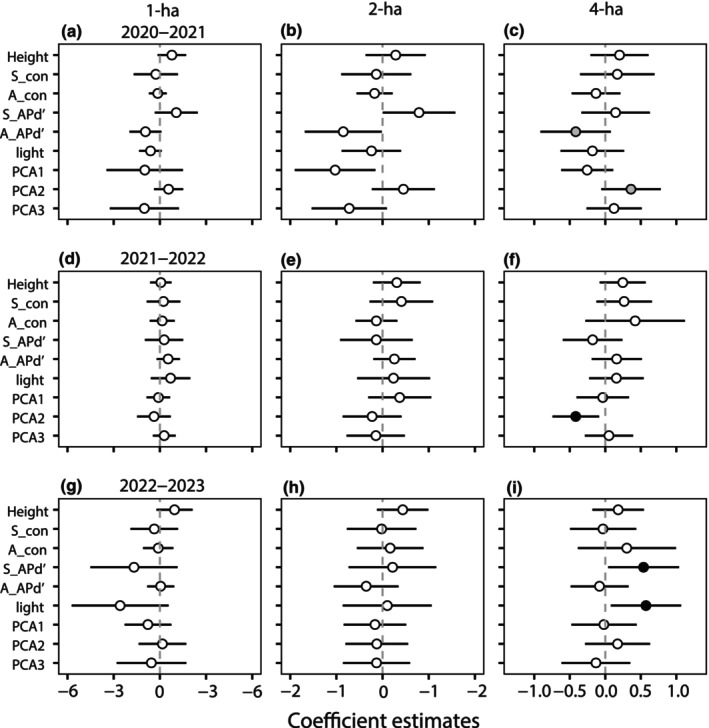
Estimated coefficients (mean ± SE) of neighbor densities and habitat variables on seedling survival of phylogenetic + habitat models at tree spatiotemporal scales. 95% Confidence intervals of the variables. The black circles indicate significant effects (*p* < .05), gray circles signify marginally significant effects (.05 < *p* < .1), and white circles mean no significance (*p* ≥ .1). See Table 1 for variable abbreviations.

The effects of phylogenetic densities for four phylogenetic indices on seedling survival differed at different spatial scales (Figure 4, Figures S2–S5, Table S5). At the 1‐ and 2‐ha scales, none of the variables had a significant effect on seedling survival at the three 1‐year intervals. However, at the 4‐ha scale, seedling survival was significantly affected by seedling APd′ in the 2022–2023 interval (Figure 4i). The effect of PCA2 on seedling survival was significant during the 2020–2022 interval (Figure 4c,f). In addition, there was a significant positive effect of light on seedling survival during the 2022–2023 interval (Figure 4i). The results showed that the effects of phylogenetic density and habitat factors on seedling survival emerged only at the largest scale. Spatial scales in neighborhood studies can influence the effects of neighbor densities and habitat variables on seedling survival.

## 4 DISCUSSION

Community phylogenetic approaches are useful for exploring community assembly mechanisms (Cavender‐Bares & Reich, 2012). Density dependence, phylogenetic density dependence, and habitat filtering are the prominent mechanisms that maintain community assembly and diversity. In this study, we examined the variance of neighborhood effects on seedling survival using different phylogenetic indices at different temporal and spatial scales. We found that phylogenetic indices affected the effects of phylogenetic density dependence and habitat filtering on seedling survival. The fitting results of the models for seedling survival were inconsistent at the different spatiotemporal scales.

### 4.1 Neighborhood effect on seedling survival in models with different phylogenetic indices

A simplistic division of neighbors into conspecific and heterospecific individuals may hide the potentially large variation in the degree to which heterospecifics are similar to focal individuals (Comita et al., 2014; Johnson et al., 2014; Piao et al., 2013; Swenson, 2013). Hence, phylogenetic relatedness among neighbors should be considered when determining neighborhood effects.

In this study, interactions among heterospecific neighbors were detected using four phylogenetic indices. Our models indicated that TOTPd in the 2020–2021 interval and APd′ in 2020–2021 and 2022–2023 intervals were significant on seedling survival, but AVEPd and NTPd′ were not significant in all models. The results of APd′ were consistent with the study of Cao et al. (2018), which calculated these four phylogenetic distance indices, and found that the models with APd′ indices had stronger support than models with other indices. However, a number of studies have reported that APd′ did not influence seedling survival in subtropical forests (Du et al., 2017; Zheng et al., 2020). The less phylogenetically conserved functional traits and habitat filtering might operate simultaneously, resulting in no correlation between phylogenetic relatedness among neighbors and seedling survival. We found that NTPd′ had no significance on seedling survival. Wang et al. (2020) concluded that the survival of seedlings was not best described by the phylogenetic + abiotic model with NTPd′ at community level in Gutianshan subtropical forest. AVEPd is the average of the sum of the phylogenetic distances between neighbors and focal seedlings. The overall similarity of an individual to its neighbors based on the mean phylogenetic distance might mask important information regarding neighborhood interactions (Chen et al., 2018).

We found positive effects of APd′ in the 2020–2021 interval and TOTPd in the 2022–2023 interval on seedling survival, indicating that increasing phylogenetic similarities between heterospecific seedling neighbors and focal seedlings caused a decrease in seedling survival (i.e., phylogenetic positive density dependence). Overall, positive phylogenetic parameter estimates indicated that seedlings surrounded by more closely related neighbors had a lower probability of survival. Density dependence among phylogenetically closely related species partially results from competition for similar resources (Burns & Strauss, 2011; Violle et al., 2011). Liu et al. (2012) found a phylogenetic Janzen–Connell effect, which may have been caused by host‐specific fungal pathogens in the Heishiding of subtropical evergreen broad‐leaved forest. Distantly related species may have positive effects if they can obstruct the spread of species‐specific pathogens of the focal species (Wills, 1996). Focal trees, living with fewer related heterospecific neighbors, are thought to suffer less from resource competition and natural enemies, and thus exhibit increased survival (Cao et al., 2018; Chen et al., 2016; Huang et al., 2022; Wu et al., 2016). The neighborhood effect, in some cases, cannot be detected which may be related to the differences in the effects of phylogenetic distance among neighborhood effect and sample size. The larger sample size, the easier it is for the effect to become apparent (Khalilzadeh & Tasci, 2017).

The effects of habitat variables varied among the models with different phylogenetic indices. Our study provides evidence that PCA2 has significant positive effects on seedling survival in the 2020–2021 interval in models with TOTPd and APd′. High C, TN, and TP levels are positively associated with seedling survival. Seedlings benefit from fertile soils (Bai et al., 2012). This result indicates a significant habitat preference for woody plants (Jin et al., 2018; Metz, 2012). The availability of topographic and soil resources is also an essential factor affecting seedling survival at the seedling stage (Comita et al., 2009; Piao et al., 2013).

However, PCA2 had a significantly negative effect on seedling survival in the 2021–2022 interval with four phylogenetic indices and the 2022–2023 interval with AVEPd. Seedling survival was lower in habitats with high C and TN and low EC, AP, AK, TK, and soil moisture. Seedling survival is higher in moist habitats (Brenes‐Arguedas et al., 2009). Fertile soils with high carbon and nitrogen are conducive to seedling survival (Lin et al., 2012; Pu et al., 2017). A host of studies have shown that light availability strongly affects seedling performance (Bai et al., 2012; Comita et al., 2009; Piao et al., 2013; Queenborough et al., 2009). The positive effect of the light on seedling survival with APd′ and NTPd′ in the 2022–2023 interval was consistent with previous studies.

Generally, we deem that the APd′ is a better phylogenetic index for seedling survival than the other three indices in this study.

### 4.2 Neighborhood effect on seedling survival with different temporal scales

Seedling survival in the Gaoligong Mountains forest exhibited marked differences in response to local neighborhoods at different temporal scales. Our findings showed that temporal scales affected the relative importance of neighbor density and habitat filtering on seedling survival. The effects of neighbor density and environmental variables on seedling survival were more likely to be revealed at the 1‐year interval since seedlings are more sensitive to these effects for a short time interval.

Our results showed that the effects of phylogenetic relatedness among neighbors on seedling survival were affected by temporal scales, and phylogenetic relatedness affected seedling survival only at 1‐year intervals, which is in agreement with previous studies (Huang et al., 2022; Wu et al., 2016). Moreover, several studies have investigated the effects of phylogenetic density dependence on seedling survival on shorter temporal scales (Cao et al., 2018; Du et al., 2017; Lu et al., 2015).

In this study, the effects of habitat variables were more obvious in the models at a 1‐year interval. In other words, the effects of habitat variables on seedling survival varied among temporal scales. There were significant effects of light and PCA2 on seedling survival at 1‐year intervals. Light availability was significantly positive for seedling survival during the 2021–2023 interval. Similar results were found in Panama (Augspurger, 1984), the Luquillo Forest (Comita et al., 2009), and temperate forests of Northeastern China (Yao et al., 2020), where higher seedling survival rates occurred at gap sites. Consequently, the effects of neighbor density and habitat filtering on seedling survival were more prominent on the 1‐year scale.

### 4.3 Neighborhood effect on seedling survival with different spatial scales

Previous studies have found an important relationship between the spatial scale and community structure (Cavender‐Bares et al., 2006; Swenson et al., 2007). Communities tended to be phylogenetically clustered with an increased spatial extent. We confirmed that the neighborhood effect on seedling survival was more obvious at a larger spatial scale (4 ha), whereas there was no significant neighborhood effect at smaller scales (1 and 2 ha). This result was similar to that of Webb et al. (2006), who found significant effects of phylogenetic density dependence at relatively larger spatial scales (4 and 36 m^2^) in a tropical forest in Southeast Asia, and no apparent effect of phylogenetic density dependence at smaller scales (0.25 and 1 m^2^). Our study also revealed that habitat factors were significant for seedling survival only on a large scale (4‐ha) and the neighborhood effect was not significant in the 2021–2022 interval (Figure 4i). With an increase in spatial scale, environmental variables gradually increase, and habitat filtering becomes the dominant force in community construction (Willis et al., 2010). Our results further support the idea that the effects of environmental factors such as elevation, topography, and soil at different spatial scales differ for different communities (Huang et al., 2010). Due to the smaller spatial scale, the effects of phylogenetic density dependence and habitat filtering on seedling survival may be obscured (Cavender‐Bares et al., 2018).

In summary, the study of the neighborhood effect was optimal at the largest spatial scale (4 ha) in a mid‐mountain moist evergreen broad‐leaved forest in the Gaoligong Mountains. It is important to emphasize that spatial scales constrain the effects of density dependence and habitat variables on seedling survival. It is necessary to further verify the results at larger scales (>4‐ha) in the future.

## 5 CONCLUSION

In the present study, we examined whether the effects of density dependence and habitat variables on seedling survival varied in models with different phylogenetic indices, spatial scales, and temporal scales. Our results revealed that the relative importance of phylogenetic relatedness among neighbors, density dependence, and habitat filtering on seedling survival was confined by phylogenetic indices, temporal scales, and spatial scales. We found that the effects of neighborhood and environmental factors on seedling survival were more obvious in the models with the phylogenetic index APd′ on a 1‐year temporal scale and the largest spatial scale (4 ha). We suggest that the phylogenetic index APd′ be preferred and a larger spatial scale should be established for timely monitoring of seedling survival in future works on neighborhood density dependence.

## AUTHOR CONTRIBUTIONS

**Liping Wang:** Conceptualization (equal); data curation (equal); formal analysis (equal); investigation (equal); methodology (lead); visualization (lead); writing – original draft (lead); writing – review and editing (equal). **Junjie Wu:** Conceptualization (lead); data curation (equal); funding acquisition (lead); methodology (equal); project administration (lead); resources (lead); supervision (lead); validation (lead); writing – review and editing (lead). **Yong Chai:** Data curation (equal); investigation (equal). **Jiwen Sun:** Data curation (equal); formal analysis (equal); investigation (equal). **Xiaoli Yu:** Data curation (equal); formal analysis (equal); investigation (equal). **Zhe Feng:** Investigation (equal). **Fengxian Chen:** Investigation (equal).

## FUNDING INFORMATION

This study was supported by the National Natural Science Foundation of China, Grant No. 31901102.

## CONFLICT OF INTEREST STATEMENT

The authors have no competing interests to declare.

### OPEN RESEARCH BADGES

This article has earned an Open Data badge for making publicly available the digitally‐shareable data necessary to reproduce the reported results. The data is available at https://datadryad.org/stash/share/h2bdltgfvsyZhH6BbrhREA0oBhIJdGB2u0F2QqjMxA.

## Supporting information


Appendix S1.

## Data Availability

The data that support the findings of this study are available from the Dryad Digital Repository: DOI: 10.5061/dryad.nk98sf80r and URL: https://datadryad.org/stash/share/h2bdltgfvsyZhH6BbrhREA0oBhIJdGB2u0F2OqjMxA.
