# Supplementary material for: Phylogenetic indices and temporal and spatial scales shape the neighborhood effect on seedling survival in a mid‐mountain moist evergreen broad‐leaved forest, Gaoligong Mountains, Southwestern China
Source: Ecol Evol. 2024 Jul 7;14(7):e11675. doi: 10.1002/ece3.11675 (PMC11227910; doi:10.1002/ece3.11675)
Supplement: Supplementary file 1 — Appendix S1. [file ECE3-14-e11675-s001.docx]

**Supplementary Materials**

Table S1. The loadings of thirteen habitat variables on the first three principal components (PCA1, PCA2 and PCA3). Values in the table indicate the eigenvector scores of each of the variables on the three PCA axes.

| Habitat variable | PCA1 | PCA2 | PCA3 |
| --- | --- | --- | --- |
| Elevation | 0.56 | -0.06 | -0.76 |
| Convexity | 0.26 | -0.06 | -0.81 |
| Slope | -0.39 | -0.19 | -0.07 |
| Soil pH | 0.22 | -0.30 | -0.48 |
| Soil electrical conductivity (EC) | 0.68 | -0.50 | 0.31 |
| Soil organic matter content (C) | 0.53 | 0.75 | 0.11 |
| Soil available phosphorus (AP) | 0.66 | -0.54 | 0.15 |
| Soil available potassium (AK) | 0.70 | -0.59 | 0.23 |
| Soil total nitrogen (TN) | 0.63 | 0.68 | 0.11 |
| Soil total phosphorus (TP) | 0.77 | 0.35 | -0.07 |
| Soil total potassium (TK) | -0.68 | -0.56 | -0.09 |
| Soil temperature | -0.31 | 0.16 | 0.02 |
| Soil moisture | 0.67 | -0.41 | 0.15 |
| Variation explained (%) | 32.59 | 20.56 | 13.00 |

Appendix S1. The equations to describe the models built in this study.

We constructed phylogenetic + habitat models as follows:

$$s_{ij}=\log\left( \frac{p_{ij}}{1-p_{ij}} \right)$$

$=\left[ \beta_{0}+{\beta_{1}Height}_{ij}+\beta_{2}{Density}_{i}+\beta_{3}{Phylo}_{i}+\beta_{4}{Habitat}_{j} \right]_{\begin{aligned} fixed\_part \end{aligned}}+ \left[ \mu_{\mathrm{species}}+\mu_{\begin{aligned} qua \end{aligned}} \right]_{random\_part}+\varepsilon_{ij}$ (eqn S1)

Where $s_{ij}$is the binary response (alive or dead) for seedling *i* in quadrat *j*, $p_{ij}$ is the predicted survival probability for each seedling. The first set of brackets in equation includes the fixed part of the model, *β*_0_ denotes the fixed intercept. *β*_1-4_ denotes the coefficient of the initial heights of focal seedlings (*Height_ij_*), neighborhood density variables (*Density_i_*), neighborhood phylogenetic variables (*Phylo_i_*), and habitat variables (*Habitat_j_*). The second set of brackets includes the random part of the model. *μ*_species_ is the species-specific random intercept. *μ_qua_* is the seedling quadrat-specific random intercept. *ε_ij_* is the [error](javascript:void(0);) term of the model for seedling *i* in quadrat *j*.

Table S2. The means and ranges of all continuous explanatory variables in Generalized linear mixed models (GLMMs).

| Fixed factor | 2020-2021 | | 2021-2022 | | 2022-2023 | |
| --- | --- | --- | --- | --- | --- | --- |
|  | Range | Mean | Range | Mean | Range | Mean |
| Height (cm) | 6.70~229.60 | 58.41 | 11.00~255.00 | 66.29 | 14.00~303.00 | 75.07 |
| S_con | 0~27 | 7.252 | 0~26 | 7.060 | 0~24 | 6.699 |
| A_con | 0.000~1.206e-03 | 4.700e-05 | 0.000~1.206e-03 | 4.515e-05 | 0~1.206e-03 | 4.732e-05 |
| Light | 2.510~10.590 | 7.036 | 2.510~10.590 | 7.048 | 2.51~10.59 | 7.06 |
| PCA1 | -3.424~6.657 | -0.262 | -3.424~6.657 | -0.225 | -3.424~6.657 | -0.269 |
| PCA2 | -6.825~3.138 | 0.132 | -6.825~3.138 | 0.154 | -6.825~3.138 | 0.151 |
| PCA3 | -4.724~7.305 | -0.122 | -4.724~7.305 | -0.099 | -7.305~4.724 | 0.067 |
| S_TOTPd | 0.000~15.455 | 1.750 | 0.000~14.704 | 1.771 | 0.000~13.954 | 1.705 |
| A_TOTPd | 0.661~64.187 | 54.554 | 0.661~64.187 | 54.653 | 0.661~64.187 | 54.659 |
| S_AVEPd | 0.000~0.817 | 0.194 | 0.000~0.817 | 0.205 | 0.000~0.817 | 0.197 |
| A_AVEPd | 0.274~0.340 | 0.305 | 0.274~0.340 | 0.305 | 0.274~0.340 | 0.304 |
| S_APd’ | -13.442~-4.077 | -7.508 | -13.757~-4.061 | -7.382 | -13.259~ -4.277 | -7.327 |
| A_APd’ | -7.031~-3.198 | -4.161 | -6.321~-3.315 | -4.351 | -6.329~ -3.379 | -4.701 |
| S_NTPd’ | -4.724~-0.969 | -2.590 | -4.779~-0.943 | -2.590 | -4.798~ -0.964 | -2.605 |
| A_NTPd’ | -3.103~-0.749 | -0.869 | -2.665~-0.748 | -0.854 | -2.988~ -0.982 | -1.056 |

Note: the height of focal seedlings (Height); the density of conspecific (S_con); the density of conspecific adult neighbors (A_con); canopy openness (Light); first principal component analysis of topographic and soil variables (PCA1); second principal component analysis of topographic and soil variables (PCA2); third principal component analysis of topographic and soil variables (PCA3); four phylogenetic distance indices: total phylogenetic distance (TOTPd), average phylogenetic distance (AVEPd), relative average phylogenetic distance (APd’) and relative nearest phylogenetic distance (NTPd’), respectively for seedling neighbors (S_TOTPd, S_AVEPd, S_APd’ and S_NTPd’) and adult neighbors (A_TOTPd, A_AVEPd, A_APd’ and A_NTPd’).

Table S3. Akaikae information criterion (AIC) and corresponding R^2^ for fixed and random effects (R^2^) values for phylogenetic + habitat models for each of two-year and three-year intervals.

| Models | 2020–2022 | | 2021–2023 | | 2020–2023 | |
| --- | --- | --- | --- | --- | --- | --- |
|  | AIC | R^2^ | AIC | R^2^ | AIC | R^2^ |
| Height + S_ con + A_con +S_TOTPd +A_ TOTPd+ light+PCA1+PCA2+PCA3 | **445.212** | 0.068 | 471.278 | 0.128 | **547.110** | 0.161 |
| Height + S_ con + A_con + S_AVEPd +A_ AVEPd+ light+PCA1+PCA2+PCA3 | 448.316 | 0.060 | 471.277 | 0.127 | **547.771** | 0.161 |
| Height + S_ con + A_con + S_APd’ +A_ APd’ + light+PCA1+PCA2+PCA3 | 448.151 | 0.054 | **469.149** | 0.126 | **546.404** | 0.160 |
| Height + S_ con + A_con + S_NTPd’ +A_NTPd’ + light+PCA1+PCA2+PCA3 | 448.127 | 0.060 | **470.415** | 0.116 | **546.869** | 0.153 |

Note: A bold font indicates that the △AIC ≤ 2. See Table S2 for variable abbreviations.

Table S4. Coefficient estimates for all explanatory variables in the phylogenetic + habitat models with four phylogenetic indices at three temporal scales.

| Explanatory variables | One-year | | | | | | | | | | | | Two-year | | | | | | | | Three-year | | | |
| --- | --- | --- | --- | --- | --- | --- | --- | --- | --- | --- | --- | --- | --- | --- | --- | --- | --- | --- | --- | --- | --- | --- | --- | --- |
|  | 2020–2021 | | | | 2021–2022 | | | | 2022–2023 | | | | 2020–2022 | | | | 2021–2023 | | | | 2020–2023 | | | |
| Height | 0.212 | 0.253 | 0.201 | 0.181 | **0.278#** | 0.262 | 0.246 | 0.264 | 0.180 | 0.189 | 0.181 | 0.194 | 0.088 | 0.084 | 0.085 | 0.079 | 0.190 | 0.190 | 0.204 | 0.194 | 0.106 | 0.100 | 0.113 | 0.105 |
| S_ con | -0.096 | 0.077 | 0.169 | 0.029 | 0.285 | 0.242 | 0.266 | 0.268 | -0.058 | -0.016 | -0.030 | -0.043 | 0.122 | 0.133 | 0.151 | 0.159 | 0.145 | 0.142 | 0.134 | 0.140 | 0.064 | 0.071 | 0.058 | 0.071 |
| A_con | -0.031 | -0.183 | -0.130 | -0.134 | 0.436 | 0.409 | 0.420 | 0.444 | 0.242 | 0.249 | 0.305 | 0.273 | 0.128 | 0.084 | 0.089 | 0.075 | 0.485 | 0.500 | 0.514 | 0.474 | 0.154 | 0.140 | 0.149 | 0.139 |
| S_TOTPd | **0.891*** |  |  |  | 0.049 |  |  |  | -0.108 |  |  |  | 0.298 |  |  |  | -0.033 |  |  |  | 0.133 |  |  |  |
| A_ TOTPd | **0.534*** |  |  |  | -0.146 |  |  |  | 0.080 |  |  |  | 0.160 |  |  |  | -0.048 |  |  |  | 0.111 |  |  |  |
| S_AVEPd |  | 0.262 |  |  |  | -0.157 |  |  |  | 0.050 |  |  |  | -0.006 |  |  |  | -0.013 |  |  |  | 0.050 |  |  |
| A_ AVEPd |  | -0.021 |  |  |  | -0.002 |  |  |  | 0.066 |  |  |  | -0.025 |  |  |  | -0.050 |  |  |  | -0.042 |  |  |
| S_APd’ |  |  | 0.145 |  |  |  | -0.178 |  |  |  | 0.540 |  |  |  | 0.010 |  |  |  | 0.177 |  |  |  | 0.201 |  |
| A_ APd’ |  |  | **-0.418#** |  |  |  | 0.162 |  |  |  | -0.080 |  |  |  | -0.112 |  |  |  | -0.049 |  |  |  | -0.050 |  |
| S_NTPd’ |  |  |  | 0.035 |  |  |  | 0.037 |  |  |  | -4.58 |  |  |  | 0.072 |  |  |  | -0.200 |  |  |  | -0.123 |
| A_NTPd’ |  |  |  | -0.082 |  |  |  | -0.035 |  |  |  | -0.085 |  |  |  | -0.018 |  |  |  | -0.200 |  |  |  | -0.130 |
| light | -0.239 | -0.186 | -0.182 | -0.166 | 0.126 | 0.211 | 0.157 | 0.199 | 0.348 | 0.356 | 0.573 | 0.534 | -0.014 | 0.010 | 0.008 | -0.005 | **0.350*** | **0.345*** | **0.419*** | **0.418*** | 0.151 | 0.168 | 0.232 | 0.185 |
| PCA1 | 0.010 | -0.329 | -0.255 | -0.180 | -0.080 | -0.089 | -0.035 | -0.051 | 0.045 | 0.064 | -0.018 | -0.015 | -0.156 | -0.241 | -0.231 | -0.225 | -0.006 | 0.011 | -0.003 | 0.040 | -0.093 | -0.140 | -0.155 | -0.109 |
| PCA2 | **0.624**** | 0.307 | **-0.362#** | 0.340 | **-0.448*** | **-0.040*** | **-0.415*** | **-0.389*** | 0.107 | 0.077 | 0.173 | 0.131 | -0.020 | -0.124 | -0.121 | -0.125 | -0.233 | -0.210 | -0.179 | -0.209 | 0.021 | -0.027 | -0.025 | -0.058 |
| PCA3 | 0.052 | 0.053 | 0.125 | 0.118 | 0.060 | 0.108 | 0.052 | 0.103 | -0.074 | -0.041 | -0.132 | -0.124 | 0.087 | 0.104 | 0.109 | 0.105 | 0.218 | 0.222 | 0.252 | **0.408#** | 0.220 | 0.226 | **0.258#** | **0.324#** |

Note: See Table S2 for variable abbreviations.

Bold values indicate ** *P*<0.01; * *P*<0.05; # 0.05<*P*<0.1.

Table S5. Coefficient estimates for all explanatory variables in the phylogenetic + habitat models for each phylogenetic index at three spatiotemporal scales.

| Explanatory variables | One-year | | | | | | | | | Two-year | | | | | | Three-year | | |
| --- | --- | --- | --- | --- | --- | --- | --- | --- | --- | --- | --- | --- | --- | --- | --- | --- | --- | --- |
|  | 2020–2021 | | | 2021–2022 | | | 2022–2023 | | | 2020–2022 | | | 2021–2023 | | | 2020–2023 | | |
|  | 1-ha | 2-ha | 4-ha | 1-ha | 2-ha | 4-ha | 1-ha | 2-ha | 4-ha | 1-ha | 2-ha | 4-ha | 1-ha | 2-ha | 4-ha | 1-ha | 2-ha | 4-ha |
| TOTPd | | | | | | | | | | | | | | | | | | |
| Height | 0.664 | 0.184 | 0.212 | 0.208 | 0.359 | **0.278#** | **0.832#** | **0.497#** | 0.180 | 0.226 | 0.335 | 0.088 | 0.359 | **0.346#** | 0.190 | 0.318 | **0.336#** | 0.106 |
| S_ con | -0.279 | -0.532 | -0.096 | 0.518 | 0.365 | 0.285 | 0.499 | 0.031 | -0.058 | -0.191 | 0.410 | 0.122 | 0.595 | 0.156 | 0.145 | 0.086 | 0.264 | 0.064 |
| A_con | -0.233 | -0.185 | -0.031 | 0.017 | -0.239 | 0.436 | 0.325 | 0.162 | 0.242 | -0.061 | 0.179 | 0.128 | 0.134 | -0.053 | 0.485 | 0.103 | 0.319 | 0.154 |
| S_TOTPd | 0.126 | 0.697 | **0.892*** | -0.051 | 0.232 | 0.049 | 0.580 | 0.002 | -0.108 | -0.377 | **0.671#** | 0.298 | 0.073 | -0.265 | -0.033 | -0.238 | 0.227 | 0.133 |
| A_ TOTPd | **1.176#** | **0.981*** | **0.534*** | 0.280 | 0.200 | -0.146 | 0.786 | 0.284 | 0.080 | 0.161 | 0.303 | 0.160 | 0.233 | 0.126 | -0.048 | 0.163 | 0.200 | 0.111 |
| light | -0.528 | -0.210 | -0.239 | 0.492 | 0.439 | 0.236 | **-1.137#** | 0.025 | 0.348 | -0.011 | -0.068 | -0.014 | **0.628#** | 0.261 | **0.350*** | 0.088 | -0.098 | 0.151 |
| PCA1 | -0.920 | -0.277 | 0.010 | -0.317 | 0.463 | -0.080 | -0.236 | -0.080 | 0.045 | -0.138 | 0.059 | -0.156 | -0.215 | 0.181 | -0.006 | -0.057 | 0.016 | -0.093 |
| PCA2 | **1.100#** | 0.918* | **0.624**** | -0.594 | -0.197 | **-0.448*** | -0.334 | -0.109 | 0.107 | 0.295 | 0.181 | -0.020 | -0.710 | -0.036 | -0.233 | 0.206 | 0.191 | 0.021 |
| PCA3 | -1.282 | -0.525 | 0.052 | 0.108 | -0.125 | 0.060 | -0.895 | -0.035 | -0.074 | 0.286 | -0.055 | 0.087 | -0.147 | 0.229 | 0.218 | 0.071 | 0.130 | 0.220 |
| AVEPd | | | | | | | | | | | | | | | | | | |
| Height | 0.647 | 0.176 | 0.253 | 0.232 | 0.379 | 0.262 | **1.065#** | **0.517#** | 0.189 | 0.260 | 0.339 | 0.084 | 0.402 | **0.357#** | 0.190 | 0.362 | **0.341#** | 0.099 |
| S_ con | -0.055 | -0.175 | 0.077 | 0.446 | 0.419 | 0.242 | 0.254 | 0.116 | -0.016 | -0.189 | 0.397 | 0.133 | 0.415 | 0.297 | 0.142 | -0.150 | 0.333 | 0.071 |
| A_con | -0.284 | -0.205 | -0.183 | -0.087 | -0.285 | 0.409 | 0.106 | 0.109 | 0.249 | -0.085 | 0.143 | 0.084 | -0.026 | -0.020 | 0.497 | -0.019 | 0.301 | 0.140 |
| S_ AVEPd | -0.276 | -0.116 | 0.262 | -0.230 | -0.210 | -0.157 | 2.310 | 0.296 | 0.050 | -0.438 | **0.635#** | -0.006 | -0.345 | -0.009 | -0.013 | -0.602 | 0.394 | 0.050 |
| A_ AVEPd | -0.043 | -0.116 | -0.021 | 0.263 | 0.280 | -0.002 | 1.267 | 0.188 | 0.066 | 0.238 | 0.338 | -0.025 | 0.051 | 0.133 | -0.050 | 0.097 | 0.204 | -0.042 |
| light | **-0.670#** | -0.174 | -0.186 | 0.489 | 0.386 | 0.211 | -0.914 | 0.028 | 0.356 | -0.101 | 0.119 | 0.010 | **0.602#** | 0.209 | **0.345*** | -0.009 | -0.020 | 0.168 |
| PCA1 | -0.841 | -0.617 | -0.329 | -0.225 | 0.563 | -0.089 | -0.864 | -0.150 | 0.064 | -0.049 | 0.009 | -0.241 | -0.090 | 0.182 | 0.011 | 0.086 | -0.041 | -0.140 |
| PCA2 | 0.069 | 0.209 | 0.307 | -0.535 | -0.165 | **-0.398*** | 0.073 | -0.129 | 0.077 | 0.238 | 0.250 | -0.124 | -0.632 | -0.099 | -0.210 | 0.185 | 0.216 | -0.027 |
| PCA3 | -0.783 | -0.508 | 0.053 | 0.201 | -0.151 | 0.108 | -0.462 | 0.011 | -0.041 | 0.213 | -0.030 | 0.104 | 0.044 | 0.114 | 0.222 | 0.121 | 0.130 | 0.226 |
| APd’ | | | | | | | | | | | | | | | | | | |
| Height | **0.778#** | 0.284 | 0.201 | 0.060 | 0.308 | 0.246 | 0.952 | 0.435 | 0.181 | 0.153 | 0.324 | 0.085 | 0.249 | 0.344 | 0.204 | 0.247 | **0.326#** | 0.113 |
| S_ con | -0.267 | -0.137 | 0.169 | 0.238 | 0.407 | 0.266 | -0.364 | -0.021 | -0.030 | -0.010 | 0.277 | 0.151 | 0.121 | 0.287 | 0.134 | 0.045 | 0.207 | 0.058 |
| A_con | -0.136 | -0.173 | -0.130 | 0.151 | -0.133 | 0.420 | -0.112 | 0.164 | 0.305 | 0.221 | 0.050 | 0.089 | 0.203 | 0.001 | 0.514 | 0.305 | 0.260 | 0.149 |
| S_ APd’ | 1.067 | **0.789*** | 0.145 | 0.268 | -0.134 | -0.178 | -1.682 | 0.214 | **0.540*** | 0.043 | -0.408 | 0.010 | 0.886 | 0.172 | 0.177 | 0.150 | -0.242 | 0.201 |
| A_ APd’ | **-0.940#** | **-0.850*** | **-0.418#** | 0.545 | 0.257 | 0.162 | 0.054 | -0.356 | -0.080 | 0.483 | 0.013 | -0.112 | 0.444 | 0.088 | -0.049 | 0.459 | 0.090 | -0.050 |
| light | -0.615 | -0.243 | -0.182 | 0.696 | 0.239 | 0.157 | -2.583 | 0.100 | **0.573*** | 0.029 | -0.115 | 0.008 | **1.382*** | 0.316 | **0.419*** | 0.196 | -0.150 | 0.232 |
| PCA1 | -0.983 | **-1.028*** | -0.255 | -0.098 | 0.372 | -0.035 | -0.772 | -0.166 | -0.018 | -0.066 | 0.050 | -0.231 | 0.048 | 0.166 | -0.003 | 0.037 | 0.005 | -0.155 |
| PCA2 | 0.564 | 0.450 | **0.362#** | -0.386 | -0.227 | **-0.415*** | 0.169 | -0.126 | 0.173 | 0.178 | 0.182 | -0.121 | -0.514 | -0.099 | -0.179 | 0.188 | 0.180 | -0.025 |
| PCA3 | -1.006 | **-0.721#** | 0.125 | 0.286 | -0.149 | 0.052 | -0.545 | -0.132 | -0.132 | 0.039 | 0.033 | 0.109 | 0.217 | 0.147 | 0.252 | -0.050 | 0.165 | **0.258#** |
| NTPd’ | | | | | | | | | | | | | | | | | | |
| Height | 0.567 | 0.466 | 0.181 | 0.154 | 0.340 | 0.264 | 0.796 | **0.499#** | 0.194 | 0.190 | 0.295 | 0.079 | 0.315 | 0.329 | 0.194 | 0.274 | 0.283 | 0.105 |
| S_ con | -0.256 | -0.438 | 0.029 | 0.738 | 0.549 | 0.268 | 0.277 | -0.021 | -0.043 | 0.805 | 0.427 | 0.159 | 0.555 | 0.354 | 0.140 | 0.796 | 0.363 | 0.071 |
| A_con | -0.272 | -0.212 | -0.134 | 0.051 | -0.176 | 0.444 | -0.077 | 0.140 | 0.273 | 0.166 | 0.058 | 0.075 | 0.110 | -0.004 | 0.474 | 0.254 | 0.223 | 0.139 |
| S_ NTPd’ | -0.947 | **-0.660#** | 0.035 | 0.338 | 0.189 | 0.037 | 1.370 | -0.074 | -0.458 | 0.602 | **0.473#** | 0.072 | -0.060 | 0.079 | -0.200 | 0.489 | 0.265 | -0.123 |
| A_ NTPd’ | 0.279 | 0.036 | -0.083 | -0.141 | -0.002 | -0.035 | -0.337 | 0.188 | -0.085 | -0.311 | -0.092 | -0.018 | -0.219 | -0.347 | -0.200 | -0.475 | -0.444 | -0.130 |
| light | **-0.612#** | 0.015 | -0.166 | 0.193 | 0.222 | 0.199 | **-2.469#** | 0.066 | **0.534*** | -0.272 | -0.101 | -0.005 | 0.655 | 0.109 | **0.418*** | -0.107 | -0.174 | 0.185 |
| PCA1 | -1.865 | -0.691 | -0.180 | -0.362 | 0.422 | -0.051 | -0.807 | -0.119 | -0.015 | -0.184 | 0.071 | -0.225 | -0.194 | 0.203 | 0.040 | -0.052 | 0.053 | -0.109 |
| PCA2 | 0.625 | 0.254 | 0.340 | -0.759 | -0.309 | **-0.389*** | -0.094 | -0.085 | 0.131 | -0.044 | 0.207 | -0.125 | **-0.723#** | -0.158 | -0.209 | -0.040 | 0.132 | -0.058 |
| PCA3 | -1.299 | -0.511 | 0.118 | 0.114 | -0.214 | 0.103 | -0.602 | -0.061 | -0.124 | 0.141 | 0.113 | 0.105 | 0.077 | 0.361 | **0.408#** | 0.144 | 0.509 | **0.324#** |

Note: See Table S2 for variable abbreviations.

Bold values indicate ** *P*<0.01; * *P*<0.05; # 0.05<*P*<0.1.


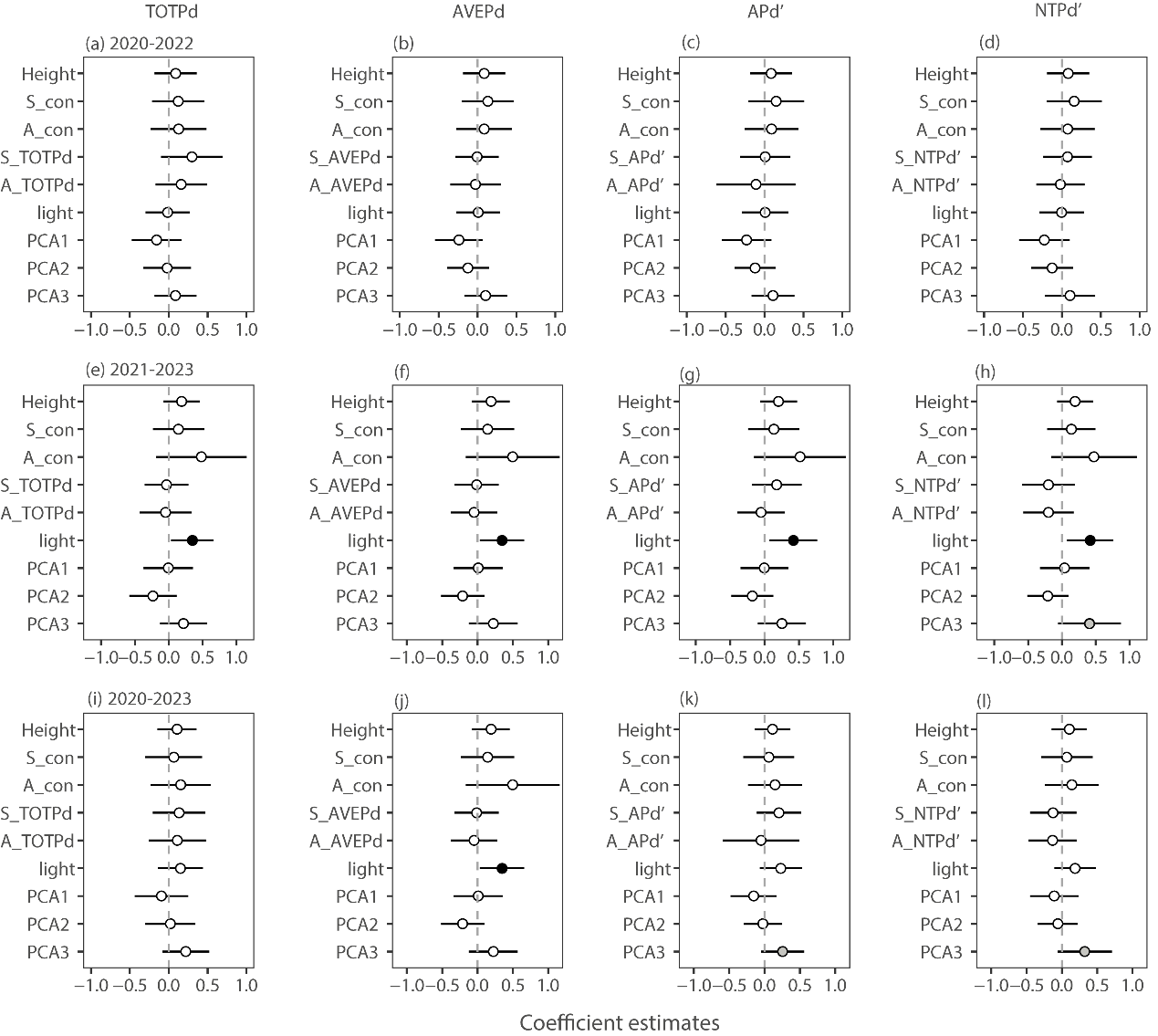


Figure S1. Estimated coefficients (mean ± SE) of neighbor densities and habitat variables on seedling survival using four phylogenetic indices of phylogenetic + habitat models at two-year and three-year temporal scales. 95% confidence intervals of the variables (Error bars represent 95% confidence intervals). The black circles indicate significant effects (*P* < 0.05), gray circles signify marginally significant effects (0.05 < *P* < 0.1), and white circles mean no significance (*P* ≥ 0.1). See Table S2 for variable abbreviations.


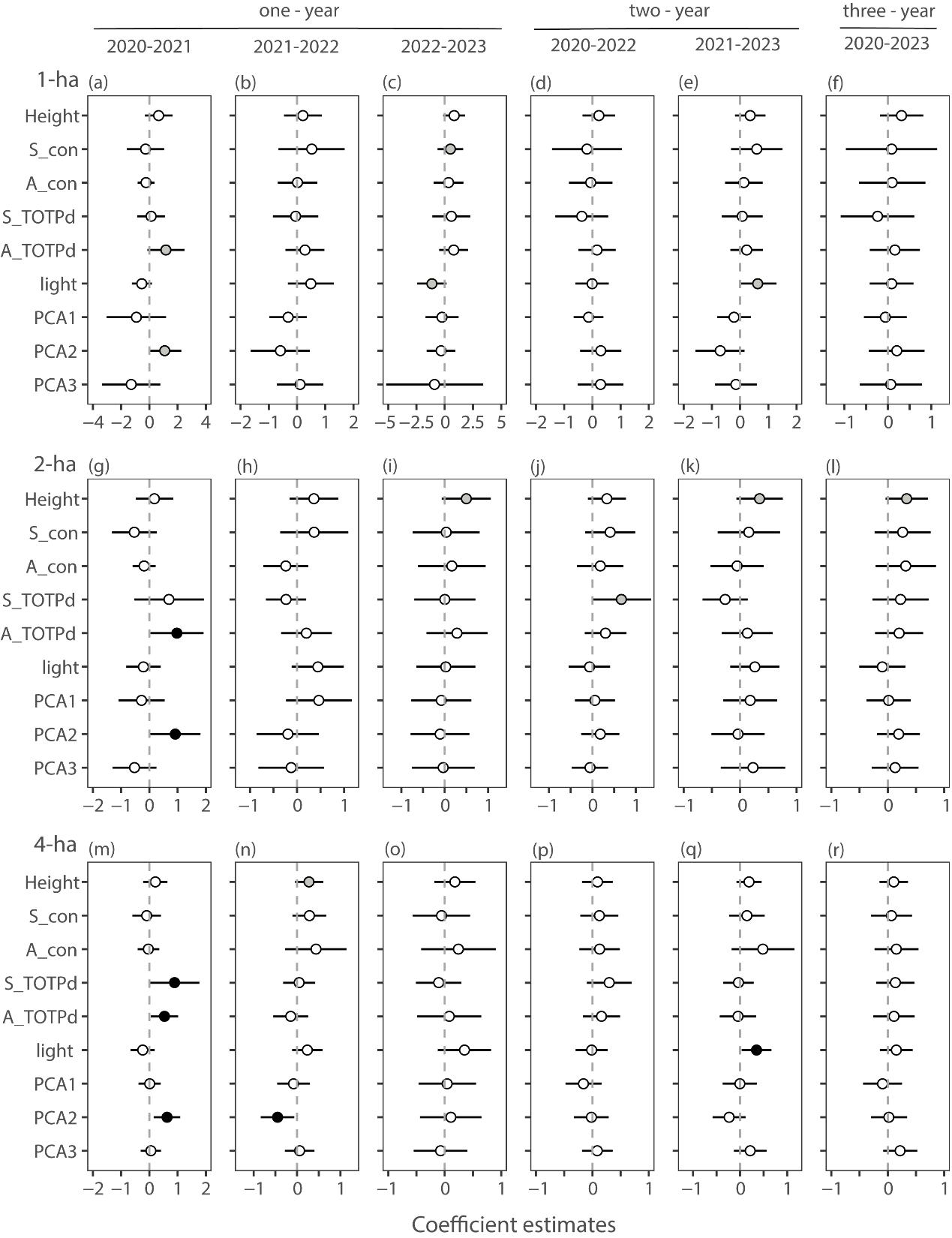


Figure S2. Estimated coefficients (mean ± SE) of neighbor densities and habitat variables on seedling survival of phylogenetic + habitat models with TOTPd at three spatiotemporal scales. 95% confidence intervals of the variables. The black circles indicate significant effects (*P* < 0.05), gray circles signify marginally significant effects (0.05 < *P* < 0.1), and white circles mean no significance (*P* ≥ 0.1). See Table S2 for variable abbreviations.


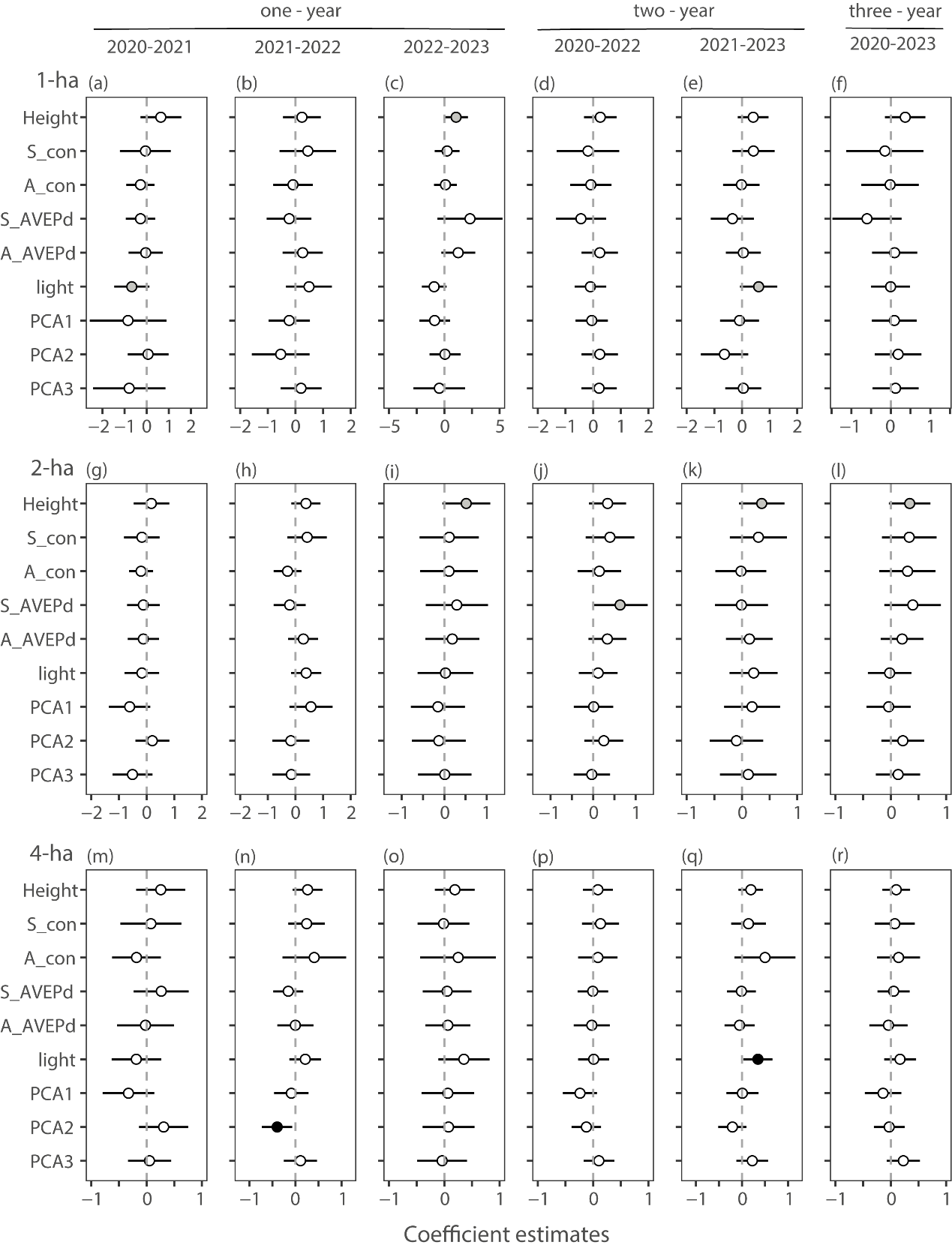


Figure S3. Estimated coefficients (mean ± SE) of neighbor densities and habitat variables on seedling survival of phylogenetic + habitat models with AVEPd at three spatiotemporal scales. 95% confidence intervals of the variables. The black circles indicate significant effects (*P* < 0.05), gray circles signify marginally significant effects (0.05 < *P* < 0.1), and white circles mean no significance (*P* ≥ 0.1). See Table S2 for variable abbreviations.


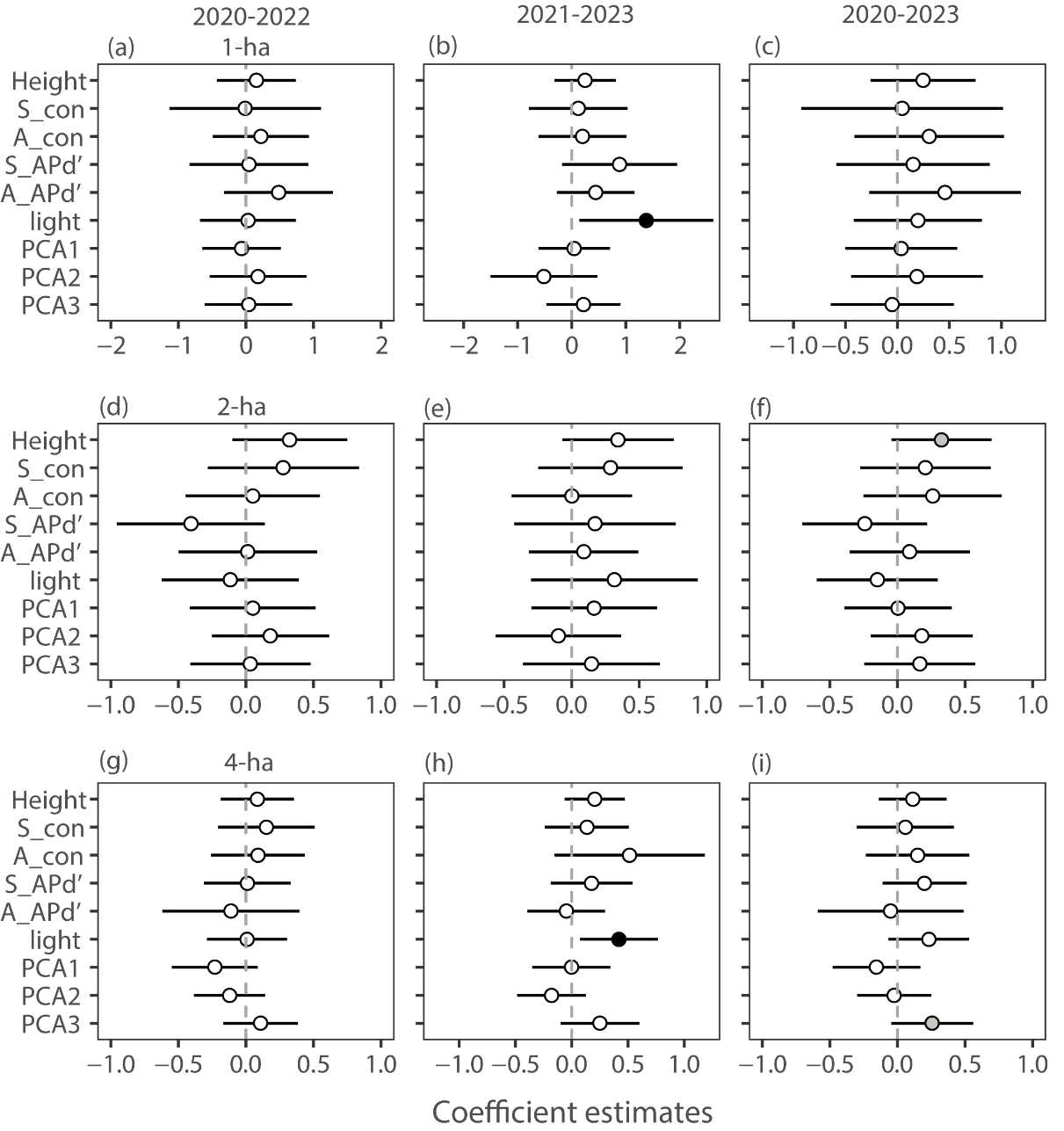


Figure S4. Estimated coefficients (mean ± SE) of neighbor densities and habitat variables on seedling survival of phylogenetic + habitat models with APd’ at three spatial scales and two temporal scales (two-year and three-year). 95% confidence intervals of the variables. The black circles indicate significant effects (*P* < 0.05), gray circles signify marginally significant effects (0.05 < *P* < 0.1), and white circles mean no significance (*P* ≥ 0.1). See Table S2 for variable abbreviations.


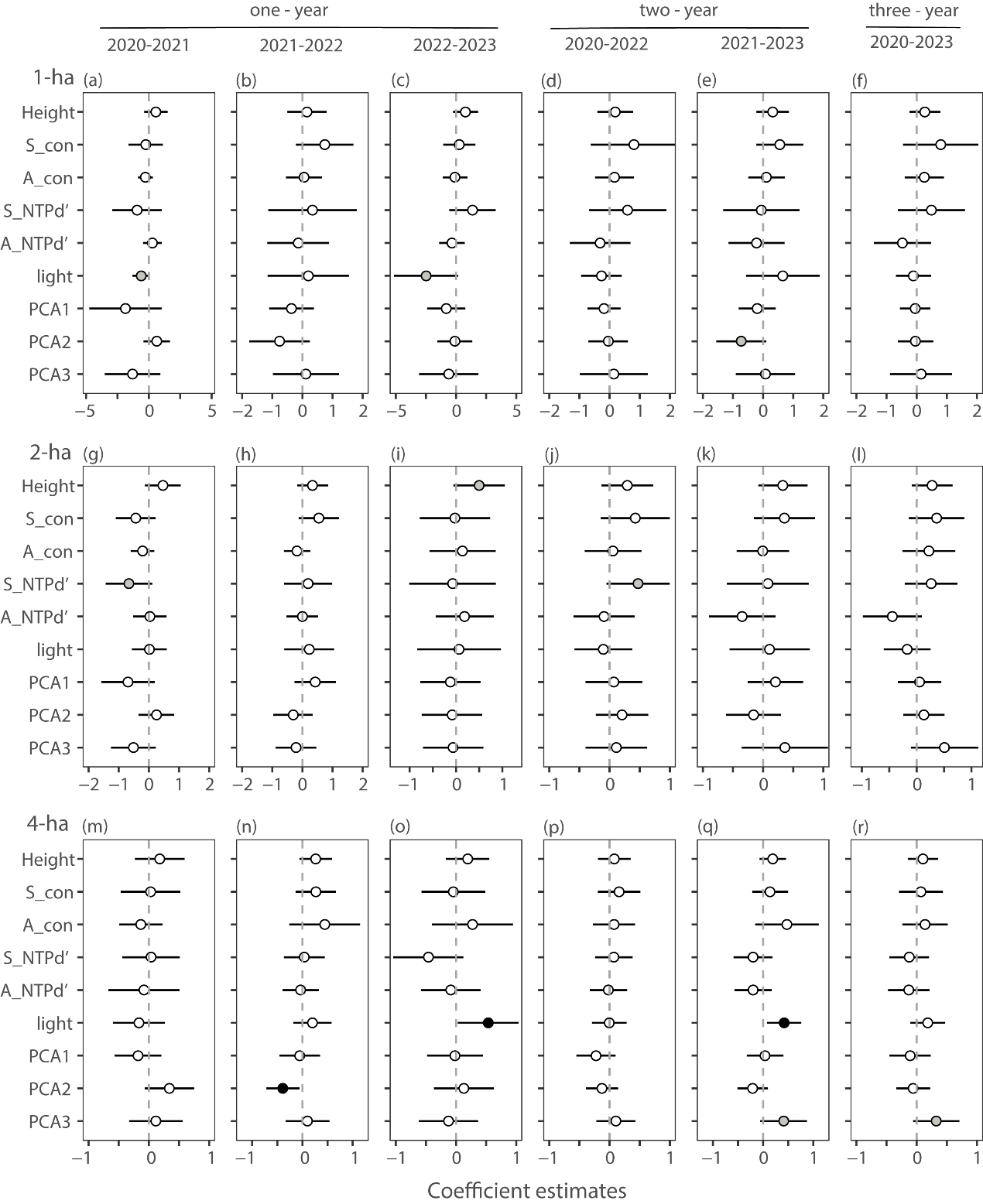


Figure S5. Estimated coefficients (mean ± SE) of neighbor densities and habitat variables on seedling survival of phylogenetic + habitat models with NTPd’ at three spatiotemporal scales. 95% confidence intervals of the variables. The black circles indicate significant effects (*P* < 0.05), gray circles signify marginally significant effects (0.05 < *P* < 0.1), and white circles mean no significance (*P* ≥ 0.1). See Table S2 for variable abbreviations.
